# Meaning and Influencing Factors of a Good Death for Community‐Dwelling Individuals With Dementia: An Integrative Review

**DOI:** 10.1111/opn.70078

**Published:** 2026-04-12

**Authors:** Jong Sun Park, Hannah Cho, Won Seok Park, Hyejin Kim

**Affiliations:** ^1^ Red Cross College of Nursing Chung‐Ang University Seoul Republic of Korea; ^2^ School of Nursing University of Pennsylvania Philadelphia Pennsylvania USA; ^3^ Subject Information Service Team Chung‐Ang University Seoul Republic of Korea

**Keywords:** community care, dementia, end of life care, good death, palliative care

## Abstract

**Introduction:**

The rising prevalence of dementia highlights the need to ensure good quality of life for those affected. This integrative review aimed to clarify what constitutes a good death for community‐dwelling people with dementia and their family caregivers and to identify structural and process factors that support or hinder its attainment.

**Methods:**

Using Whittemore and Knafl's integrative review approach, a literature search was conducted in PubMed, CINAHL, Embase, Scopus and Web of Science using key terms, including dementia, community‐dwelling and death. Following the screening process, 21 articles were analysed using the structure–process–outcome framework.

**Results:**

Of the 21 articles included, nine explored the concept of good death, 16 addressed structural factors, and 19 examined process factors associated with quality end‐of‐life care that may facilitate good death. From the perspective of people with dementia and their family caregivers, a good death involves dying peacefully while preserving dignity and autonomy. Structural factors included community‐based healthcare and social support resources, such as access to hospice and palliative care, a skilled professional workforce and services that support daily living and caregiver needs. Process factors included comprehensive approaches to patient care: advance care planning, symptom management, person‐centred care, emergency and safety management, health management and education for family caregivers.

**Conclusions:**

This review highlights that achieving a good death for community‐dwelling people with dementia requires accessible, comprehensive, person‐centred end‐of‐life care and robust support for family caregivers. Strengthening policies and developing integrated community care models are essential for upholding end‐of‐life dignity and honouring care preferences.

**Implications for Practice:**

These findings suggest that healthcare professionals should implement community‐based, person‐centered care models that provide structured education and psychological support for family caregivers to facilitate a ‘good death’ for people with dementia.

## Summary

**What does this research add to existing knowledge in gerontology?:**

- The review findings expand the existing knowledge in gerontology by clarifying the essential, multifaceted nature of a good death in dementia, as defined by both individuals with dementia and their family caregivers in community settings.
- By identifying the key structural and process factors that support quality end‐of‐life care and a good death in community settings, this review provides a conceptual foundation for developing dementia‐specific, community‐based palliative care strategies that uphold dignity, autonomy and caregiver well‐being.

**What are the implications of this new knowledge for nursing care for and with older adults?:**

- The review findings underscore the critical importance of person‐centred, community‐based end‐of‐life care that respects the dignity and autonomy of older adults with dementia.
- Improving access to community‐based hospice and palliative care services is essential for ensuring the continuity of end‐of‐life care for older adults with dementia.
- Providing tailored education and ongoing practical support to family caregivers is crucial to enable their confident participation in end‐of‐life care and facilitate a good death.

**How could the findings be used to influence practice, education, research, and policy?:**

- The findings can inform policy by advocating expanded access to community‐based hospice and palliative care services and promoting investment in a well‐trained healthcare workforce.
- In practice, they support the development of integrated care models that prioritize person‐centred approaches and caregiver support.

## 1 Background

Globally, more than 55 million people have dementia (World Health Organization 2023). As the disease progresses, people lose the ability to effectively communicate their health concerns and care preferences, leading to challenges in delivering care that meets their needs (National Academies of Sciences and Medicine 2021). Additionally, addressing the care needs of people with end‐stage dementia who are non‐communicative, predominantly bedridden and completely dependent on others for basic daily living activities (such as eating, dressing and ambulating) places a substantial burden on family caregivers (Song et al. 2023). Furthermore, uncertainty regarding the wishes of people with dementia frequently imposes emotional strain on family caregivers, contributing to feelings of helplessness and guilt (Caputo 2021). Thus, high‐quality end‐of‐life care that addresses the profound impact of dementia should be made available to people with this condition and their family caregivers.

Quality end‐of‐life care for people with chronic illnesses involves effective management of pain and symptoms, avoidance of life‐prolonging interventions, preservation of personal agency, relief of burden and support for meaningful relationships with loved ones (Shepperd et al. 2021). These characteristics align with those of a good death identified in various studies exploring the perspectives of patients, family caregivers and healthcare providers (Mamun et al. 2023; Meier et al. 2016). A good death is commonly associated with physical comfort (being pain‐free), psychological and emotional well‐being, a degree of control over the dying process and the ability to maintain normalcy as long as possible (Houska and Loučka 2019). Given its deeply personal and dynamic nature, the concept of a good death must be explored from the perspective of people with dementia and their family caregivers to provide quality end‐of‐life care tailored to their needs (Meier et al. 2016).

Consistent with the concept of a good death, many people with dementia express a preference for dying at home; however, a significant proportion die in acute care hospitals (Donnelly et al. 2018) or nursing homes (Eisenmann et al. 2020). Reliance on institutional end‐of‐life care reflects the limited availability and capacity of community‐based services (Hoare et al. 2019). Common barriers to delivering such care to people with life‐limiting illnesses and their family caregivers include the low availability of home‐based care services, shortage of trained care providers and palliative care specialists, poor coordination among care teams, insufficient knowledge and skills among healthcare providers and families and inadequate education and training in palliative and end‐of‐life care (Gupta and Patel 2024; Lalani and Cai 2022). However, the under‐recognition of dementia as a terminal illness, uncertainty regarding the end‐of‐life process, limited communication ability in people with dementia and the presence of behavioural psychological symptoms present unique challenges for people with dementia and their families in maintaining home‐based care according to their preferences (Harrison et al. 2019). Furthermore, people with dementia often receive lower‐quality end‐of‐life care than those with cancer (Martinsson et al. 2021).

Thus, this review aims to comprehensively examine and synthesize findings from peer‐reviewed scientific studies to elucidate the meaning of a good death, its facilitators and challenges from the perspectives of community‐dwelling people with dementia and family caregivers. We used Donabedian's structure–process–outcome model to identify the facilitators and challenges related to the structure and process of quality end‐of‐life care that contribute to a good death.

## 2 Materials and Methods

### 2.1 Design

This integrative review followed Whittemore and Knafl's (2005) approach to synthesize the results of quantitative, qualitative and mixed method studies on good death and community end‐of‐life care in the context of dementia. This approach involves five steps: problem identification (aims), literature search, data evaluation, data analysis and presentation of results.

### 2.2 Literature Search

#### 2.2.1 Search Strategy

The literature search strategy was developed in consultation with a librarian. Two authors (JSP and HC) independently searched PubMed, Cumulative Index in Nursing and Allied Health Literature, Embase, Scopus and the Web of Science. The following key terms were used in the database search: (a) dementia, Alzheimer's disease, frontotemporal dementia, dementia, vascular disease and Lewy body disease; (b) independent living, home environment and community dwelling; and (c) death, dying, good death, terminal care and palliative care (Table S1). The search was conducted on April 24, 2024, with no restrictions on the publication period.

#### 2.2.2 Eligibility Criteria

The eligibility criteria for study selection were structured according to the PICO‐SD (Population, Intervention, Comparison, Outcome and Study Design) framework: (a) Population: Studies focussing on community‐dwelling people with dementia or their family caregivers were included, whereas studies targeting care providers or those conducted exclusively in nursing homes or hospitals were excluded; (b) Intervention (interest): Factors influencing end‐of‐life care or conceptualizations of a good death constituted the primary area of interest; (c) Comparison: No specific comparison group was applicable; (d) Outcome: No pre‐specified outcomes were defined; and (e) Study Design: Original research employing quantitative, qualitative or mixed method designs was included, whereas reviews, research protocols, case studies and comments/editorials were excluded. Only the articles published in English were included.

#### 2.2.3 Study Selection Process

**FIGURE 1:**
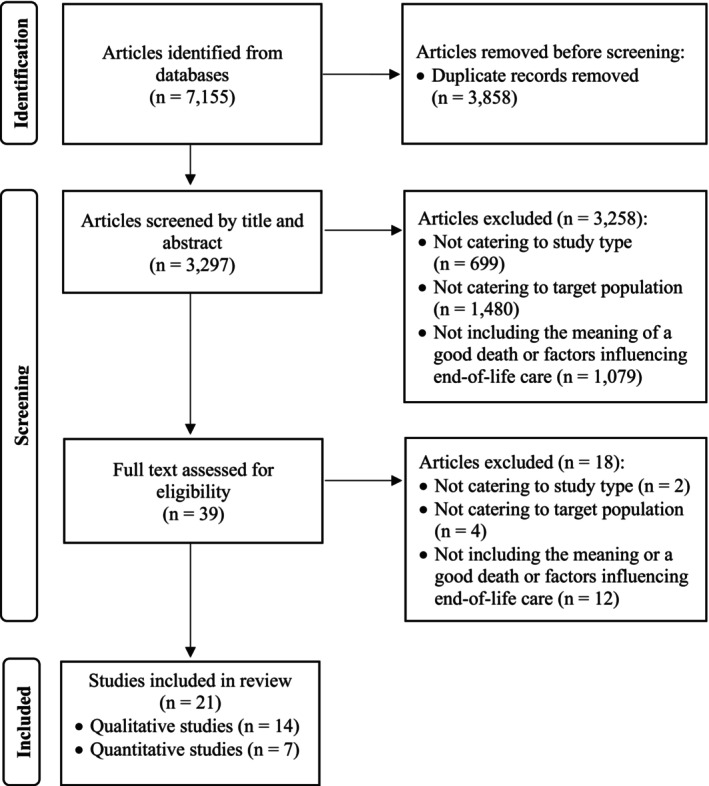
Flow chart of the participant selection process.

Two authors (JSP and HC) independently screened the titles and abstracts based on the inclusion criteria. Full texts were subsequently reviewed for eligibility and studies that met the exclusion criteria were excluded. Discrepancies at each stage were resolved through discussions within the research team. The study selection process is shown in Figure 1.

### 2.3 Data Evaluation

Two authors (JSP and HC) independently assessed the quality of the 21 included studies using the mixed methods appraisal tool (Hong et al. 2018). The tool consists of a five‐item checklist for each study type (qualitative study, randomized controlled trial, non‐randomized trials, quantitative descriptive study and mixed methods), with response options of ‘Yes’, ‘No’ and ‘Can't tell’. The evaluation results of each author were compared and discussed within each group to resolve any discrepancies. No study was excluded based on the quality assessment results.

### 2.4 Data Analysis

Two authors (JSP and HC) independently extracted data from the 21 included studies using a pre‐structured Excel spreadsheet that consisted of the study objectives, design, participants, settings, data collection, analysis and results related to outcomes (good death), structure and process. The extracted data were compared, and discrepancies were resolved through group discussion.

**FIGURE 2:**
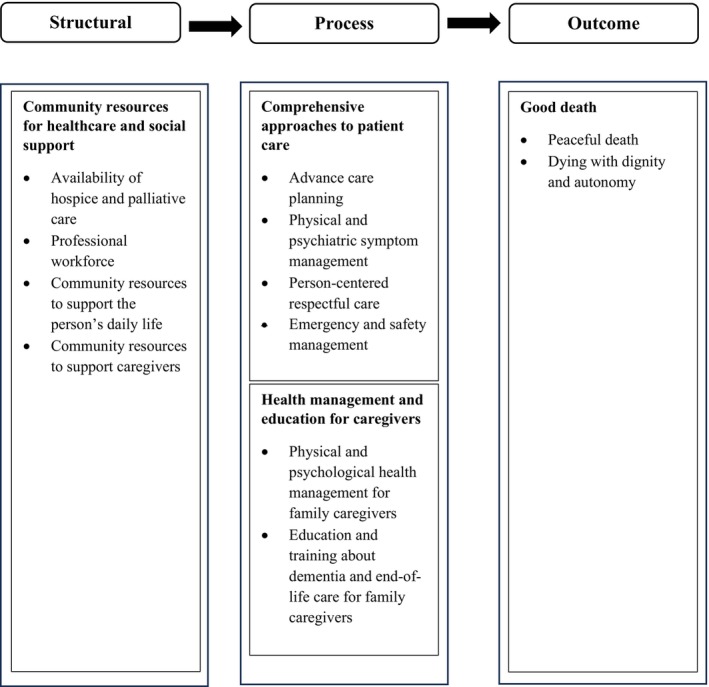
Meaning and influencing factors of A good death for community‐dwelling people with dementia. The figure presents the findings organized according to three domains (structure, process, and outcome). Within each domain, the identified categories (in bold) and their corresponding subcategories are displayed.

Directed content analysis was used to analyse data across three domains: good death, structure and process (Hsieh and Shannon 2005). To explore the meaning of a good death, two authors independently conducted line‐by‐line open coding of the relevant data, grouping similar codes into subcategories and further abstracting them into broader categories. The data extracted in the ‘structure’ and ‘process’ domains were open‐coded with a focus on identifying facilitators and challenges. As a result, many facilitators and challenges have emerged as opposing elements of the same concept (e.g., the presence or absence of advance care planning). Thus, we developed subcategories by combining contrasting factors related to a good death. The findings of this review are presented using the structure–process–outcome model (Figure 2).

## 3 Results

### 3.1 Study Selection

A literature search of electronic databases yielded 7155 articles. After removing duplicates, two authors (JSP and HC) independently screened the titles and abstracts of 3358 articles based on the inclusion criteria. Discrepancies were discussed until a consensus was reached, resulting in the selection of 39 articles for full‐text review. All authors independently reviewed the full texts for eligibility and excluded 18 articles because of inappropriate study type (*n* = 2), target population (*n* = 4) or study focus (*n* = 12). Consequently, the final sample included 14 qualitative and 7 quantitative studies (Figure 1).

### 3.2 Characteristics of the Included Studies

The final sample included 14 qualitative and 7 quantitative studies (6 non‐randomized trials and 1 descriptive study) from the United States (*n* = 6), Europe (*n* = 10) and Asia (*n* = 5). These studies included 322,811 community‐dwelling individuals with dementia (age range: 6–322,461; *n* = 4) and 1258 family caregivers (age range: 5–377; *n* = 14). Table 1 provides the detailed characteristics of the included studies.

**TABLE 1:** Summary of all included articles (*N* = 21).

Author(s) (year)/country	Objectives	Study design	Participants	Data collection and analysis	Results		
					Definition of good death	Structure	Process
Bolt et al. (2022)/Netherlands	Explore the perspectives of people with dementia regarding dependence on others in the present and future	Qualitative study (qualitative description)	18 people with dementia	Face‐to‐face, semi‐structured interviews Inductive content analysis	Maintaining dignity	Facilitators—availability of resources for engaging in meaningful activities and maintaining social connections	Facilitators—preparing for the end of life (advance care planning) and building a trustful, empathetic care relationship with professional caregivers Challenges—fear of losing autonomy and confronting cognitive decline hindered future planning and end‐of‐life discussions
Bosco et al. (2023)/United Kingdom	Explore views of bereaved family caregivers and hospice clinicians on hospice dementia care	Qualitative study (design unspecified)	Five bereaved family caregivers and seven hospice clinicians	Semi‐structured, qualitative interviews over the phone Thematic analysis	Not stated	Facilitators—availability of hospice and palliative care services Challenges—limited access to hospice and palliative care services (family caregivers' limited knowledge of hospice and palliative care services; insufficient home‐based hospice care services)	Facilitators—preparing for end‐of‐life care that reflects the patient's preferences; person‐centred and respectful attitudes of healthcare professionals (compassionate care creating a sense of peace); bereavement support for family caregivers
de Jong et al. (2023)/ Netherlands	Explore views of bereaved family caregivers, general practitioners and case managers on factors and circumstances helpful for remaining at home until the end of life	Qualitative Study (design unspecified)	11 bereaved family caregivers of people with dementia, 2 general practitioners and 9 case managers	Individual, semi‐structured interviews Thematic analysis	Remaining at home at the end of life	Facilitators—Support from a case manager, general practitioners, and/or home care nurses; wider social networks (e.g., relatives, friends, and neighbours); day‐care services	Facilitators – psychological health management for family caregivers (resilience of the family caregivers); education and training for family caregivers to support caregiving; safety management Challenges—acute, unexpected situations; dementia‐related behavioural issues; safety concerns; and caregiving burden
Dekker and Bolt (2022)/Netherlands	Explore experiences of end‐of‐life care planning	Qualitative study (secondary analysis using a naturalistic interpretative approach)	18 people with dementia and 32 bereaved family caregivers	In‐depth, semi‐structured interviews and ethnographic fieldwork Secondary thematic analysis	Not stated	Not stated	Facilitators—advance care planning (understanding the person's explicit wishes) Challenges—avoiding advance care planning as a coping mechanism for an unsettling future; lack of knowledge about when to apply the person's known care wishes (no resuscitation, no hospitalization, etc.); insufficient understanding of the consequences of treatment decisions
Dempsey et al. (2020)/Ireland	Describe family caregivers' experience of providing end‐of‐life care to people with late‐stage dementia at home	Qualitative study (phenomenology)	23 family caregivers of people with late‐stage dementia	Semi‐structured interviews Interpretative phenomenological analysis	Home as an ideal place for achieving a good death Presence of family members Pain‐free death	Facilitators—accessing formal support and services; informal caregiving support (from family) Challenges—lack of informal caregiving support; lack of social support	Challenges—not discussing death and dying early in the disease trajectory; unpredictable, fluctuating symptoms; caregiving burden and conflicting roles; lack of information about the disease process and end‐of‐life signs and symptoms
Han et al. (2019)/United States	Identify challenges, possible resources for resilience and expected consequences	Qualitative study (design unspecified)	39 family caregivers of people with dementia	Interviews Theory‐driven, deductive content analysis	Not stated	Challenges—lack of financial support and lack of cooperation and communication between family members	Facilitators–caregiver support through emotional self‐appraisal, self‐care practices and effective caregiving strategies Challenges—lack of knowledge about managing emotional stress and caregiving tasks; increased caregiving burden; caregivers' physical and mental health issues
Hossain et al. (2022)/United Kingdom	Explore the family caregivers' experiences from the initial signs of dementia to the end of life	Qualitative study (design unspecified)	16 family caregivers of people with dementia who originated from South Asia	Face‐to‐face semi‐structured interviews Thematic analysis	Not stated	Challenges—lack of formal support for people with dementia and family caregivers	Challenges—lack of information about dementia; stigma surrounding dementia and help‐seeking; inadequate and disrespectful care services due to cultural ignorance; lack of advance care planning
Jennings et al. (2017)/United States	Explore the important goals of care for people with dementia	Qualitative study (design unspecified)	Seven people with early‐stage dementia and 36 family caregivers	Focus‐group interviews Deductive and inductive coding approaches	Maintaining a good quality of life throughout the disease trajectory (engaging in meaningful activity and living at home) No burden on family at the end of life Dying peacefully	Facilitators – access to community resources that meet caregiver needs (e.g., legal and financial assistance); availability of adequate professional caregivers; respite care; social support (e.g., a support group)	Facilitators—caregiver support, managing caregiving stress, providing culturally appropriate dementia care and community‐based dementia education
Lawrence et al. (2011)/United Kingdom	Define good end‐of‐life care and identify how it can be delivered across care settings	Qualitative study (design unspecified)	27 bereaved family caregivers of people with dementia and 23 care professionals	In‐depth interviews Constant‐comparison analytic method	Enabling family members to be present at the time of death	Not stated	Facilitators—advance care planning and knowledge on how to prepare for end‐of‐life care at home
Livingston et al. (2010)/United Kingdom	Identify common difficult decisions made by family caregivers and facilitators and barriers to making such decisions	Qualitative study (design unspecified)	72 informal caregivers of people with dementia	Focus‐group and individual interviews Thematic content analysis	Not stated	Not stated	Facilitators—providing dementia‐related information to family caregivers for end‐of‐life care decisions and advance care planning while the patient retains capacity Challenges—lack of information about dementia
Malhotra and Balasubramanian (2023)/Singapore	Assess caregivers' end‐of‐life care goals for people with dementia and changes in these goals over time	Prospective cohort study	215 caregivers of people with dementia	Key variables: Caregivers' end‐of‐life care goals; change in caregivers' goals	Not stated	Not stated	Facilitators—acceptance of the patient's lower quality of life and advance care planning discussions were associated with a goal of minimal life extension Challenges—caregivers' anticipatory grief was associated with a goal of maximal life extension
Malhotra, Hazirah, et al. (2021)/Singapore	Describe suffering and its impact on their decisions	Qualitative study (design unspecified)	27 family caregivers of people with severe dementia	In‐depth, semi‐structured interviews Reflexive thematic analysis	No suffering	Not stated	Facilitators—effective symptom management Challenges—patients' unresolved discomfort or distress
Malhotra, Mohamad, et al. (2021)/Singapore	Explore factors influencing caregivers' preferences for potentially life‐extending treatments for people with severe dementia	Qualitative study (design unspecified)	26 family caregivers of people with severe dementia	In‐depth, semi‐structured interviews Reflexive thematic analysis	Being pain free	Not stated	Facilitators—implementing goals‐of‐care discussions to support end‐of‐life decision‐making Challenges—family caregivers' moral distress about making decisions to withhold, misunderstanding of end‐of‐life treatments and feeling powerless in making decisions
Mamun et al. (2023)/Japan	Explore a good death for people with dementia	Qualitative study (design unspecified)	12 people with dementia and 22 family caregivers	Focus‐group and individual interviews Inductive content analysis	Painless death Dying in a preferred environment Family's coping with loss Maintaining a regular life Living with respect Preparation for death	Facilitators—availability of resources that support engagement in preferred activities	Facilitators—advance care planning and family support Challenges—physical and emotional discomfort and conflicting preferences between a person with dementia and family caregivers
McDarby et al. (2023)/United States	Identify end‐of‐life caregiving challenges and strategies to address those challenges	Qualitative study (design unspecified)	40 bereaved family caregivers of people with dementia	Semi‐structured interviews Thematic analysis	Not stated	Facilitators—accepting external support, including paid caregivers and hospice team members	Facilitators—accepting disease progression, using pragmatic coping strategies and dementia and end‐of‐life care information Challenges—difficulty maintaining family caregiver well‐being and the lack of knowledge and information regarding dementia and caregiving skills
Mitchell et al. (2004)/United States	Describe and compare the end‐of‐life experience of people dying with advanced dementia in the nursing home and home care settings	Quantitative study (retrospective cohort study)	290 people with dementia	Key variables: health service utilization, advance care planning, non‐palliative interventions, signs and symptoms and other treatments	Not stated	Challenges—limited access to home hospice services	Challenges—insufficient advance care planning and pain and oxygen therapy and limited recognition of the end‐of‐life stage leading to delayed or missed hospice care
Oh et al. (2023)/United States	Examine the association between advanced care planning involvement, hospice utilization and hospitalizations	Quantitative study (prospective cohort study)	322,461 decedents with dementia	Key variables: advanced practice clinician involvement, hospitalization and hospice enrolment	Not stated	Facilitators—extensive advanced practice clinician involvement leading to fewer hospitalizations in the last 30 days of life and more hospice enrolment Challenges—low involvement of advanced practice clinicians leading to more hospitalizations and less hospice enrolment	Not stated
Sampson et al. (2018)/United Kingdom	Describe physical and psychological symptoms, healthcare and social service utilization and end‐of‐life care	Quantitative study (prospective cohort study)	85 people with dementia from 14 nursing homes or their own homes	Key variables: physical and psychological symptoms, quality of life and comfort, healthcare utilization and clinical events and interventions	Not stated	Facilitators—use of outpatient services and palliative care team involvement Challenges—lack of dementia and palliative care specialists	Not stated
Sternberg et al. (2014)/Israel	Examine the quality of end‐of‐life (EOL) care	Quantitative study (retrospective cohort study)	117 family caregivers of people with dementia	Key variables: symptom management at end of life in dementia and comfort assessment during dying	Not stated	Challenges—limited availability of home hospice services (provided to 8% older people)	Facilitators—family caregivers' physical and emotional capacity Challenges—poor symptom (pain, shortness of breath, fear, skin breakdown and resistance to care) management
Tay et al. (2022)/Singapore	Identify factors influencing caregiver burden and its impact on end‐of‐life care decisions	Quantitative study (prospective cohort study)	377 family caregivers of people with advanced dementia	Key variables: caregiving burden and environmental and social support factors	Not stated	Facilitators—enrolment in a home‐based palliative care service Challenges—absence of informal paid help leading to family caregivers' burden	Challenges—severe neuropsychiatric symptoms and clinical instability of people with advanced dementia
Volicer et al. (2003)/United States	Examine end‐of‐life care for people with dementia across various care settings	Quantitative study (retrospective survey)	156 family caregivers of people with dementia	Key variables: care setting, satisfaction with care, symptom management and comfort in dying	Dying at home while receiving end‐of‐life care	Facilitators—access to home hospice care	Facilitators—advance care planning and symptom management Challenges—unmanaged psychiatric symptoms and caregiving burden

### 3.3 Quality Appraisal Results

Table 2 presents the results of the quality assessment (Data S1 presents item‐level assessment results per study). Among the 14 qualitative studies, three demonstrated the appropriateness of the qualitative approach in addressing the research questions. For the remaining 11, we were unable to evaluate appropriateness because of the absence of a statement regarding the qualitative tradition employed (e.g., qualitative description, grounded theory and phenomenology). Nonetheless, all 14 studies met the following criteria: adequate data collection methods; validity of the results derived from the data; appropriate data interpretation; consistency across data sources; and collection, analysis and interpretation. For the non‐randomized controlled trials (*n* = 6), most quality assessment items were fulfilled, including representativeness of the study participants (*n* = 4), appropriateness of measurements for outcomes and interventions (*n* = 6), completeness of outcome data (*n* = 6), accountability of confounders (*n* = 5) and adherence to intended interventions (*n* = 6). However, one study faced difficulties in participant recruitment and did not explicitly mention methods for controlling for confounders. Another study lacked sufficient details on the inclusion and exclusion criteria, reasons for non‐participation and efforts to ensure representativeness. One quantitative descriptive study met all but one quality assessment criterion with a high risk of non‐response bias due to a 27% response rate. These findings indicate that most of the included studies demonstrated adequate methodological quality. A small number of studies exhibited methodological limitations, including a high risk of non‐response bias. These limitations were documented and considered during the synthesis process.

**TABLE 2:** Quality appraisal results of all included studies.

Criteria	Yes	No	Cannot tell
Quality appraisal results of qualitative studies (*N* = 14)			
Is the qualitative approach appropriate to answer the research question?	3	0	11
2 Are the qualitative data collection methods adequate to address the research question?	14	0	0
3 Are the findings adequately derived from the data?	14	0	0
4 Is the interpretation of results sufficiently substantiated by data?	14	0	0
5 Is there coherence between qualitative data sources, collection, analysis, and interpretation?	14	0	0
Quality appraisal results of non‐randomized control trials (*N* = 6)			
Are the participants representative of the target population?	4	1	1
2 Are measurements appropriate regarding both the outcome and intervention (or exposure)?	6	0	0
3 Are there complete outcome data?	6	0	0
4 Are the confounders accounted for in the design and analysis?	5	1	0
5 During the study period, is the intervention administered (or exposure occurred) as intended?	6	0	0
Quality appraisal results of quantitative descriptive study (*N* = 1)			
Is the sampling strategy relevant to address the research question?	1	0	0
2 Is the sample representative of the target population?	1	0	0
3 Are the measurements appropriate?	1	0	0
4 Is the risk of nonresponse bias low?	0	1	0
5 Is the statistical analysis appropriate to answer the research question?	1	0	0

### 3.4 Meaning of a Good Death and Its Influencing Factors

For community‐dwelling people with dementia and their family caregivers, a good death is defined as a comfortable and peaceful death that upholds dignity and autonomy. The structure‐ and process‐related factors that appeared to facilitate quality end‐of‐life care, thereby promoting a good death, included community resources for healthcare and social support, comprehensive approaches to patient care and health management and education for family caregivers. The absence of these factors seemed to hinder the quality of end‐of‐life care and death (Figure 2).

#### 3.4.1 Meaning of Good Death

Nine studies included in this review examined the concept of a good death (Bolt et al. 2022; de Jong et al. 2023; Dempsey et al. 2020; Jennings et al. 2017; Lawrence et al. 2011; Malhotra, Hazirah, et al. 2021; Malhotra, Mohamad, et al. 2021; Mamun et al. 2023; Volicer et al. 2003). One study explored the concept from the perspective of people with dementia (Bolt et al. 2022), six focussed on the family caregivers' perspectives (de Jong et al. 2023; Dempsey et al. 2020; Lawrence et al. 2011; Malhotra, Hazirah, et al. 2021; Malhotra, Mohamad, et al. 2021; Volicer et al. 2003), and two examined both perspectives (Jennings et al. 2017; Mamun et al. 2023). Based on these studies, a good death for people with dementia and their family caregivers is characterized by a comfortable and peaceful transition while maintaining dignity and autonomy.

##### 3.4.1.1 Peaceful Death

Across eight studies, a ‘peaceful death’ encompassed various aspects, such as ‘death without pain’ (Dempsey et al. 2020; Jennings et al. 2017; Malhotra, Hazirah, et al. 2021; Malhotra, Mohamad, et al. 2021; Mamun et al. 2023), ‘death surrounded by family’ (Dempsey et al. 2020; Lawrence et al. 2011) and ‘death at home’ (de Jong et al. 2023; Dempsey et al. 2020; Volicer et al. 2003). Specifically, in two studies, people with dementia expressed a desire to pass away without experiencing pain (Jennings et al. 2017; Mamun et al. 2023), surrounded by their loved ones at the end of their life (Mamun et al. 2023), and without becoming a burden on them (Jennings et al. 2017), in a familiar and comfortable environment such as their own home (Mamun et al. 2023). Similarly, in four studies, family caregivers emphasized the importance of ensuring that their loved ones had a comfortable and pain‐free end of life (Dempsey et al. 2020; Malhotra, Hazirah, et al. 2021; Malhotra, Mohamad, et al. 2021) and were physically present to provide emotional comfort as an essential element of a good death (Dempsey et al. 2020; Lawrence et al. 2011). Furthermore, caregivers respect the wish of the person to die at home (de Jong et al. 2023) and demonstrate their dedication by preparing the necessary care settings, such as home‐based hospital beds and caregiving services (Lawrence et al. 2011; Mamun et al. 2023).

##### 3.4.1.2 Dying With Dignity and Autonomy

Three studies reported that, along with peaceful death, people with dementia and their family caregivers perceived good death as one in which dignity and autonomy were preserved (Bolt et al. 2022; Jennings et al. 2017; Mamun et al. 2023). For them, such death entails honouring the individual's preferences and choices regarding end‐of‐life care and ensuring that dignity is upheld until the end (Jennings et al. 2017; Mamun et al. 2023). People with dementia wish to make decisions about aspects of their death, and family caregivers agree that such decisions should be respected (Jennings et al. 2017; Mamun et al. 2023). Both groups hoped to maintain their daily routines for as long as possible before death (Jennings et al. 2017; Mamun et al. 2023). In particular, people with dementia emphasize the importance of engaging in meaningful activities at the end of their lives, such as hobbies at home, walking, reading newspapers and participating in structured programmes (Bolt et al. 2022).

#### 3.4.2 Structure: Community‐Based Resources for Healthcare and Social Support

Sixteen studies identified structural factors that contribute to the quality of end‐of‐life care and facilitate the death of people with dementia and their family caregivers (Bolt et al. 2022; Bosco et al. 2023; de Jong et al. 2023; Dempsey et al. 2020; Han et al. 2019; Hossain et al. 2022; Jennings et al. 2017; Mamun et al. 2023; McDarby et al. 2023; Mitchell et al. 2004; Oh et al. 2023; Sampson et al. 2018; Sternberg et al. 2014; Tay et al. 2022; Volicer et al. 2003). These factors are related to the availability of community‐based healthcare and social support resources that provide end‐of‐life care. Key resources include hospice and palliative care services, healthcare professionals who provide essential services and various community‐based support systems that assist people with dementia and their family caregivers in their daily lives.

##### 3.4.2.1 Availability of Community‐Based Hospice and Palliative Care Services

Seven studies reported that access to hospice and palliative services within communities is essential to ensure quality end‐of‐life care for people with dementia and their family caregivers (Bosco et al. 2023; McDarby et al. 2023; Mitchell et al. 2004; Sampson et al. 2018; Sternberg et al. 2014; Tay et al. 2022; Volicer et al. 2003). In two studies, home‐based palliative care services facilitated early care planning, which eased caregiving responsibilities (Tay et al. 2022), thereby enabling people with dementia to remain at home for longer and increasing the likelihood of death (Volicer et al. 2003). Despite these benefits, access to these services is limited in many communities (Mitchell et al. 2004; Sternberg et al. 2014). For example, Sternberg et al. (2014) reported that only 8% of people with advanced dementia in Israel received home‐based hospice and palliative care.

##### 3.4.2.2 Supportive Professional Workforce

In four studies, support from healthcare professionals, including advanced practice nurses and formal caregivers, enabled people with dementia to remain at home during their final days (de Jong et al. 2023; Jennings et al. 2017; Oh et al. 2023; Sampson et al. 2018). Oh et al. (2023) reported that the greater involvement of healthcare professionals in community‐based end‐of‐life care was associated with lower hospitalization rates and increased utilization of hospice services among this population. Similarly, de Jong et al. (2023) reported that community‐based professionals play a key role in assisting family caregivers in providing long‐term care for people with dementia at home. However, a shortage of dementia and palliative care specialists in many communities has limited the availability of appropriate end‐of‐life care (Oh et al. 2023) and impeded effective symptom management and palliative care for people with dementia (Sampson et al. 2018).

##### 3.4.2.3 Community Resources to Support the Person's Daily Life

Three studies demonstrated that day care centres and home health services helped people with dementia engage in meaningful activities and maintain social connections throughout their lives (Bolt et al. 2022; Hossain et al. 2022; Mamun et al. 2023). However, accessing community‐based resources remains challenging. Hossain et al. (2022) found that barriers such as long waiting times, limited support, high costs, and language barriers often prevent people with dementia from engaging in daily activities and maintaining social relationships.

##### 3.4.2.4 Community Resources to Support Caregivers

In six studies, family caregivers consistently reported the need for legal, financial and human resources to reduce the physical and psychological burdens of caring for people with dementia at home (de Jong et al. 2023; Dempsey et al. 2020; Han et al. 2019; Hossain et al. 2022; Jennings et al. 2017; Tay et al. 2022). The lack of such support often results in delays in care coordination and financial strain (Han et al. 2019; Hossain et al. 2022). Moreover, limited manpower and financial resources often make it difficult for family caregivers to temporarily step away from their responsibilities and care for their well‐being (Dempsey et al. 2020). However, Hossain et al. (2022) reported that some caregivers (particularly those lacking structural resources) perceived caregiving as an inevitable familial duty that continued despite substantial hardship (Hossain et al. 2022). In Jennings et al. (2017), family caregivers identified social support through dementia care groups as important for maintaining emotional resilience and providing care at home.

#### 3.4.3 Process: Comprehensive Approaches to Patient Care

Of the 19 studies examining process factors contributing to quality end‐of‐life care and enabling a good death, 16 highlighted the importance of comprehensive person‐centred care (Bolt et al. 2022; Bosco et al. 2023; Dekker and Bolt 2022; Dempsey et al. 2020; Han et al. 2019; Hossain et al. 2022; Jennings et al. 2017; Lawrence et al. 2011; Livingston et al. 2010; Malhotra and Balasubramanian 2023; Malhotra, Hazirah, et al. 2021; Malhotra, Mohamad, et al. 2021; Mamun et al. 2023; McDarby et al. 2023; Mitchell et al. 2004; Volicer et al. 2003). This approach involves advanced care planning, physical and psychiatric symptom management, person‐centred respectful care and emergency and safety management for people with dementia.

##### 3.4.3.1 Advance Care Planning

In nine studies, people with dementia and their family caregivers highlighted the critical importance of advanced care planning before a person with dementia loses their decision‐making capacity to ensure that their wishes are respected (Bosco et al. 2023; Dekker and Bolt 2022; Han et al. 2019; Hossain et al. 2022; Lawrence et al. 2011; Livingston et al. 2010; Malhotra and Balasubramanian 2023; Malhotra, Mohamad, et al. 2021; Mamun et al. 2023). Such planning not only enables people with dementia to receive care aligned with their preferences and experience death consistent with their values (Bosco et al. 2023; Volicer et al. 2003) but also provides psychological relief to family caregivers by reducing uncertainty about care directions (Bosco et al. 2023; Dekker and Bolt 2022). Lawrence et al. (2011) showed that early and well‐structured advance care planning enabled family caregivers to fulfill their wishes to die at home by facilitating access to home‐based end‐of‐life care services and preparing care settings. However, Dekker and Bolt (2022) reported that engaging in advance care planning remains emotionally challenging for both parties because discussions about death and dying are often perceived as difficult and distressing. Family caregivers often attribute the lack of advance care planning to limited or ineffective communication with healthcare providers and insufficient information about care (Han et al. 2019).

##### 3.4.3.2 Physical and Psychiatric Symptom Management

Eight studies have demonstrated that managing symptoms such as pain, dyspnoea, anxiety and depression is essential to preserving the quality of life of people with dementia in their final stages (Dempsey et al. 2020; Malhotra, Hazirah, et al. 2021; Mamun et al. 2023; McDarby et al. 2023; Mitchell et al. 2004; Sternberg et al. 2014; Tay et al. 2022; Volicer et al. 2003). Pain, dyspnoea and depression are common among home‐dwelling individuals with advanced dementia (Mitchell et al. 2004). Volicer et al. (2003) reported that appropriate interventions, such as medications, oxygen therapy, pain management and psychiatric care, enable people with dementia to remain at home for longer periods. However, several individuals with dementia do not receive adequate symptom management (Malhotra, Hazirah, et al. 2021; Mamun et al. 2023; Sternberg et al. 2014; Tay et al. 2022; Volicer et al. 2003). Dempsey et al. (2020) showed that because the condition of people with dementia varies daily, their families often face significant uncertainty in managing these changes. In a study by Mamun et al. (2023), some family caregivers perceived that a lack of symptom management could significantly reduce a person's quality of life and prevent them from dying comfortably at home. Furthermore, Mitchell et al. (2004) reported that among home‐dwelling individuals with advanced dementia, caregivers recognized a life expectancy of < 6 months in only 15.6% of individuals, and only 13.1% received hospice care.

##### 3.4.3.3 Person‐Centred, Respectful Care

In six studies, people with dementia and their family caregivers emphasized the importance of person‐centred care (Bolt et al. 2022; Bosco et al. 2023; Hossain et al. 2022; Jennings et al. 2017; Lawrence et al. 2011; Livingston et al. 2020). Such care is fostered by establishing trustworthy and empathetic relationships between healthcare professionals and people with dementia (Bolt et al. 2022) and by healthcare professionals displaying sincere attitudes, such as kindness, respect and treating individuals with dignity (Bosco et al. 2023). In a study by Hossain et al. (2022), family caregivers also emphasized the importance of care that respects personal, religious, and sociocultural values (Hossain et al. 2022). Bosco et al. (2023) reported that family caregivers who experienced person‐centred care through hospice services felt reassured that their family members with dementia would receive appropriate care. Furthermore, hearing healthcare professionals' perspectives helped family caregivers make medical decisions that aligned with the person's best interests (Livingston et al. 2020).

##### 3.4.3.4 Emergency and Safety Management

Three studies demonstrated that people with dementia frequently encounter emergency and safety issues due to acute health problems or unexpected behaviours, and their family caregivers find it challenging to manage them effectively (de Jong et al. 2023; Dempsey et al. 2020; Mitchell et al. 2004). de Jong et al. (2023) reported that acute health events, such as emergency department visits and hospitalizations due to conditions such as acute pneumonia, combined with significant safety risks such as falls, wandering or elopement behaviour, increased the caregiving burden on family caregivers. These cumulative strains often lead to decisions on institutional care (e.g., nursing home placement), ultimately preventing people with dementia from remaining at home for the rest of their lives (de Jong et al. 2023).

#### 3.4.4 Process: Health Management and Education for Family Caregivers

As process factors contributing to quality end‐of‐life care, 10 studies highlighted the importance of health management and education for family caregivers (Bosco et al. 2023; de Jong et al. 2023; Dempsey et al. 2020; Han et al. 2019; Jennings et al. 2017; Livingston et al. 2010; Malhotra and Balasubramanian 2023; McDarby et al. 2023; Sternberg et al. 2014; Volicer et al. 2003). The absence of these factors hinders the effective management of the needs of end‐of‐life dementia patients, making caregiving at home increasingly challenging (de Jong et al. 2023; Dempsey et al. 2020; Han et al. 2019; Livingston et al. 2010; Malhotra, Mohamad, et al. 2021; McDarby et al. 2023; Volicer et al. 2003).

##### 3.4.4.1 Physical and Psychological Health Management for Family Caregivers

Several studies have reported that family caregivers often bear heavy caregiving burdens on people with dementia (de Jong et al. 2023; Dempsey et al. 2020; Volicer et al. 2003), and experience moral dilemmas when making decisions about end‐of‐life care (Malhotra, Mohamad, et al. 2021). Han et al. (2019) and Jennings et al. (2017) demonstrated that family caregivers experienced significant physical and psychological stress throughout the caregiving process and sought emotional support and counselling to alleviate these challenges. Family caregivers have adopted various strategies to manage stress, including seeking emotional support from hospice professionals, using coping mechanisms such as mindfulness and self‐reassurance, and taking short breaks from caregiving whenever possible (Han et al. 2019; McDarby et al. 2023). McDarby et al. (2023) noted that these approaches supported family caregivers in managing emotional strain and maintaining well‐being during end‐of‐life caregiving.

##### 3.4.4.2 Education and Training About Dementia and End‐of‐Life Care for Family Caregivers

In four studies, family caregivers reported difficulty providing adequate care to people with dementia because of their lack of knowledge about dementia and end‐of‐life care (Han et al. 2019; Jennings et al. 2017; Livingston et al. 2010; McDarby et al. 2023). This lack of understanding has led to misconceptions about the effects of treatments and has created uncertainty in decision‐making, making caregivers overly reliant on healthcare providers (Malhotra, Mohamad, et al. 2021). McDarby et al. (2023) reported that family caregivers who accessed education and information on dementia and end‐of‐life care found these resources helpful for managing challenges and preparing to meet their needs. Han et al. (2019) found that educational support helped family caregivers manage anxiety and provide better care.

## 4 Discussion

This integrative review explored the concept of good death and identified structural and process‐related factors shaping the quality of end‐of‐life care and good death for community‐dwelling people with dementia and their family caregivers. The analysis of 21 studies yielded four main findings: (a) good death was defined as a peaceful death with dignity and autonomy; (b) community‐based healthcare and social support resources played a crucial role in facilitating a good death in the community; (c) people with dementia required comprehensive healthcare approaches, including advance care planning, symptom management, person‐centred care and emergency and safety management; and (d) family caregivers needed health management support and education regarding dementia and end‐of‐life care to ensure a good death for the person with dementia. Notably, the wish to die at home is at the centre of our findings.

A good death for people with dementia and their family caregivers means dying peacefully at home with dignity, autonomy and the family present. This understanding aligns with the concept of a good death seen in other populations, including those with terminal illness; nursing home residents; and people with advanced heart disease or cancer (Gomes et al. 2013; Houska and Loučka 2019). The common characteristics of a good death across various patient populations include being pain‐free, experiencing spiritual comfort, dying at home, maintaining social connections, being surrounded by loved ones, avoiding burdening family and preserving dignity and autonomy (Gomes et al. 2013; Houska and Loučka 2019). For people with serious illnesses and their family caregivers, the home represents more than a physical setting; it symbolizes familiarity, relational closeness, comfort and a sense of control (Dekkers 2009). Thus, the preference for dying at home may be an expression of autonomy, dignity and peacefulness rather than as a distinct concept. However, a recent study found that, despite such a preference, most terminally ill people and family caregivers accepted that the actual place of death may depend on disease progression, symptom severity and available support (Pollock et al. 2024).

Despite similarities in the meaning of a good death, people with dementia face additional challenges in achieving it owing to progressive cognitive decline, impaired communication and often prolonged disease trajectories (Livingston et al. 2020). Achieving a good death for people with dementia may necessitate early advance care planning while cognitive function remains intact, thus establishing a framework to guide family caregivers once communication is lost. Additionally, dementia‐specific end‐of‐life care approaches are essential for interpreting non‐verbal cues and behavioural expressions that may indicate discomfort.

This review found that people with dementia and their family caregivers face significant challenges in accessing end‐of‐life care resources in their communities. These challenges include the lack of palliative care services, a shortage of the healthcare workforce and insufficient supportive resources for both people with dementia and family caregivers. The lack of palliative care services prevents people with dementia from receiving specialized symptom management and holistic end‐of‐life care, often resulting in unnecessary hospitalization and reduced quality of life (Chiang and Kao 2022; Sampson et al. 2018; Sutradhar et al. 2017). Furthermore, the scarcity of community‐based healthcare professionals hinders access to timely, appropriate and high‐quality end‐of‐life care (Haines et al. 2018; National Guideline 2019). The lack of comprehensive community support systems such as social services, respite care and psychosocial support for family caregivers further exacerbates the burden on people with dementia and family caregivers (Abe et al. 2020; Dowd et al. 2023).

The United Kingdom's 2008 end‐of‐life care strategy and Japan's 2006 regional comprehensive care system addressed these challenges by improving access to community resources and palliative care (Britain 2011; Saunders 2008; Tsutsui 2014). In South Korea, the Integrative Care Support Act of 2024 has established a foundation for delivering integrated healthcare and supportive care services within communities (National Law Information Center 2024). Under this Act, community‐dwelling people with dementia and their family caregivers can access resources by developing an integrated regional care network and expanding the community‐based workforce. Therefore, systematic enhancement and strong policy support, such as incentivizing healthcare systems to incorporate community‐based resources and care coordinators, are essential to expand the availability of palliative care services, strengthen the healthcare workforce and increase supportive resources for people with dementia and family caregivers.

Our review showed that a comprehensive end‐of‐life care approach includes advanced care planning, physical and psychiatric symptom management, person‐centred respectful care and emergency and safety management. A comprehensive and personalized end‐of‐life care approach tailored to an individual's healthcare needs and preferences is essential for people with dementia (Bosco et al. 2023; Hossain et al. 2022; Mamun et al. 2023; Volicer et al. 2003). Healthcare providers need the skills to conduct timely advanced care planning, manage symptoms, handle emergencies and safety issues and uphold the autonomy and dignity of people with dementia. Therefore, it is essential to integrate palliative care education with professional healthcare training programmes.

Healthcare management and education for family caregivers were critical components of quality end‐of‐life care and death. Healthcare professionals can play a crucial role in managing the physical and psychological well‐being of family caregivers by providing structured interventions, such as regular health checkups, stress management education and mental health counselling (García‐Vivar et al. 2024; Hopwood et al. 2018; Marziali et al. 2005). These educational programmes for caregivers could enhance caregiving competency and skills that support their physical and psychological well‐being while providing end‐of‐life care for people with dementia in communities (Becqué et al. 2019; García‐Vivar et al. 2024).

Building on the findings of this review, future research should focus on how the concept of good death can be translated into practice. Longitudinal studies can help clarify how needs and preferences change over time. Additionally, intervention‐related research is important for developing and evaluating community‐based person‐centred care models that reflect these evolving needs. Such studies could focus on translating the key facilitators identified in this review into community‐based approaches that support good death for people with dementia and their family caregivers. Facilitating good death aligns with the principles of improving planetary health by reducing unnecessary resource‐intensive medical interventions, thereby decreasing the environmental footprint of end‐of‐life care.

### 4.1 Strengths and Limitations

To our knowledge, this is the first integrative review to summarize and describe the concept of a good death and its structure‐ and process‐related factors from the perspectives of community‐dwelling people with dementia and family caregivers. This study offers valuable insights and practical implications for advancing community‐based dementia and end‐of‐life care through future research, policies and practice. However, this study has a few limitations. First, despite a comprehensive search strategy, we may have missed relevant studies, particularly non‐English or non‐indexed studies, limiting the cultural diversity in the findings of this review. Second, this review was limited to peer‐reviewed scientific literature and excluded perspectives from caregiver memoirs, philosophical writings and other narrative sources. Consequently, the dimensions of good death with dementia that remain empirically underexplored may be underrepresented. Third, many studies have used different definitions and metrics for key concepts, which complicates the synthesis of evidence and affects the consistency of our analysis. Finally, the methodological quality of the included studies varied considerably with some limitations. Because we included all eligible studies regardless of their quality, we carefully considered how these limitations might have influenced our findings. We interpreted the findings from studies with a high risk of non‐response bias with particular caution to avoid overemphasizing or overgeneralizing the results.

## 5 Conclusion

Through a systematic process of coding and abstraction guided by the structure–process–outcome model, our analysis of the 21 included studies yielded four main findings. Achieving a good death for community‐dwelling people with dementia and family caregivers requires access to quality end‐of‐life care supported by community‐based care resources, a comprehensive person‐centred care approach and adequate health management and education for family caregivers. To strengthen community‐based end‐of‐life care, it is essential to improve and develop healthcare policies, expand support for family caregivers and establish integrated and personalized community care models. Enhanced community‐based end‐of‐life care allows people with dementia and their family caregivers to remain in their preferred settings and maintain their dignity until the final moments of life.

## Author Contributions

J.S.P. contributed to conceptualization, methodology, data collection, formal analysis and writing of the original draft. H.C. contributed to methodology, interpretation of the findings and writing of the original draft, manuscript review and editing. H.K. contributed to methodology, interpretation of the findings, supervision and critical revision/editing of the manuscript. W.S.P. contributed to methodology, manuscript review and editing. All authors contributed to the interpretation of the findings and approved the final manuscript.

## Funding

This work was supported by the National Institute of Nursing Research (grant no. T32NR009356).

## Conflicts of Interest

The authors declare no conflicts of interest.

## Supporting information


**Table S1:** Example of search strategy.

**Table S2:‐1.** Methodological quality appraisal results of qualitative studies.
**Table S2:‐2** Methodological quality appraisal results of quantitative non‐randomized studies.
**Table S2:‐3** Methodological quality appraisal results of quantitative descriptive studies.

## Data Availability

Data sharing not applicable to this article as no datasets were generated or analysed during the current study.
