# Supplementary material for: Meaning and Influencing Factors of a Good Death for Community‐Dwelling Individuals With Dementia: An Integrative Review
Source: Int J Older People Nurs. 2026 Apr 12;21(3):e70078. doi: 10.1111/opn.70078 (PMC13070890; doi:10.1111/opn.70078)
Supplement: Supplementary file 1 — Table S1: Example of search strategy. [file OPN-21-e70078-s002.docx]

**Supplementary File 1**

**Table S1. Example of search strategy**

| **No.** | **Database** | **Search terms** |
| --- | --- | --- |
| 1 | PubMed | (“Dementia”[Mesh] OR Dementia*[TIAB] OR “Alzheimer Disease”[Mesh] OR “Alzheimer Disease*”[TIAB] OR “Frontotemporal Dementia”[Mesh] OR “Frontotemporal Dementia*”[TIAB] OR “Dementia, Vascular”[Mesh] OR “Dementia*, Vascular”[TIAB] OR “vascular dementia*”[TIAB] OR “Lewy Body Disease”[Mesh] OR “Lewy Body Disease”[TIAB] OR “Lewy body dementia”[TIAB]) AND (“Independent Living”[Mesh] OR “independent living”[TIAB] OR “Home Environment”[Mesh] OR “home environment*”[TIAB] OR “aging in place”[TIAB] OR “ageing in place”[TIAB] OR community[TIAB] OR “community dwelling”[TIAB]) AND (“Death”[Mesh] OR Death[TIAB] OR “Terminal Care”[Mesh] OR “terminal care”[TIAB] OR “Palliative Care”[Mesh] OR “palliative care”[TIAB] OR dying[TIAB] OR “End of life”[TIAB] OR “well dying”[TIAB] OR “good death”[TIAB] OR “dignified death”[TIAB]) |
| 2 | EMBASE | ('dementia'/de OR 'amentia':ti,ab OR 'dementia':ti,ab OR 'demention':ti,ab OR dementias:ti,ab OR 'alzheimer disease'/de OR 'alzeimer disease':ti,ab OR 'alzeimer`s disease':ti,ab OR 'alzeimers disease':ti,ab OR 'alzheimer dementia':ti,ab OR 'alzheimer disease':ti,ab OR 'alzheimers disease':ti,ab OR 'alzheimer fibrillary change':ti,ab OR 'alzheimer fibrillary lesion':ti,ab OR 'alzheimer neurofibrillary change':ti,ab OR 'alzheimer neurofibrillary degeneration':ti,ab OR 'alzheimer neuron degeneration':ti,ab OR 'alzheimer perusini disease':ti,ab OR 'alzheimer sclerosis':ti,ab OR 'alzheimer syndrome':ti,ab OR 'alzheimer`s disease':ti,ab OR 'cortical sclerosis, diffuse':ti,ab OR 'dementia, alzheimer':ti,ab OR 'diffuse cortical sclerosis':ti,ab OR 'late onset alzheimer disease':ti,ab OR 'alzheimer diseases':ti,ab OR 'frontotemporal dementia'/de OR 'ftd (frontotemporal dementia)':ti,ab OR 'ftld':ti,ab OR 'pick complex':ti,ab OR 'pick`s complex':ti,ab OR 'dementia, frontotemporal':ti,ab OR 'frontal dementia':ti,ab OR 'frontal lobe dementia':ti,ab OR 'frontotemporal dementia':ti,ab OR 'frontotemporal dementias':ti,ab OR 'frontotemporal lobar degeneration':ti,ab OR 'multiinfarct dementia'/de OR 'dementia, multi-infarct':ti,ab OR 'dementia, multiinfarct':ti,ab OR 'dementia, vascular':ti,ab OR 'lacunar dementia':ti,ab OR 'multi-infarct dementia':ti,ab OR 'multi-infarction dementia':ti,ab OR 'multiinfarct dementia':ti,ab OR 'multiinfarction dementia':ti,ab OR 'vascular dementia':ti,ab OR 'vascular dementias':ti,ab OR 'diffuse lewy body disease'/de OR 'dlb (dementia with lewy bodies)':ti,ab OR 'dlbd':ti,ab OR 'lbd (lewy body disease)':ti,ab OR 'lewy body dementia':ti,ab OR 'lewy body dementias':ti,ab OR 'lewy body disease':ti,ab OR 'lewy body diseases':ti,ab OR 'dementia with lewy bodies':ti,ab OR 'dementia with lewy body':ti,ab OR 'diffuse lewy body disease':ti,ab) AND ('independent living'/de OR 'aging in place':ti,ab OR 'independent living':ti,ab OR 'home environment'/de OR 'home environment':ti,ab OR 'home environments':ti,ab OR 'ageing in place':ti,ab OR 'community'/de OR 'community':ti,ab OR 'community organisation':ti,ab OR 'community organization':ti,ab OR 'community dwelling':ti,ab) AND ('death'/de OR 'death':ti,ab OR 'demise':ti,ab OR 'lethal outcome':ti,ab OR 'mors':ti,ab OR 'terminal care'/de OR 'eol care':ti,ab OR 'end-of-life care':ti,ab OR 'terminal care':ti,ab OR 'palliative therapy'/de OR 'palliation':ti,ab OR 'palliative care':ti,ab OR 'palliative consultation':ti,ab OR 'palliative medicine':ti,ab OR 'palliative radiotherapy':ti,ab OR 'palliative surgery':ti,ab OR 'palliative therapy':ti,ab OR 'palliative treatment':ti,ab OR 'symptomatic treatment':ti,ab OR 'dying'/de OR 'dying':ti,ab OR 'dying patient':ti,ab OR 'moribund':ti,ab OR 'patient, dying':ti,ab OR 'end of life'/de OR 'well dying':ti,ab OR 'good death'/de OR 'dignified death':ti,ab) |
| 3 | CINAHL | ((MH "Dementia+" OR MH "Alzheimer's Disease" OR MH "Frontotemporal Dementia+" OR MH "Dementia, Vascular+" OR MH "Lewy Body Disease" OR TI (Dementia* OR "Alzheimer Disease*" OR "Frontotemporal Dementia*" OR "vascular dementia*" OR "dementia, vascular" OR "Lewy body dementia") OR AB (Dementia* OR "Alzheimer Disease*" OR "Frontotemporal Dementia*" OR "vascular dementia*" OR "dementia, vascular" OR "Lewy body dementia")) AND (MH "Community Living+" OR MH "Home Environment" OR MH "Aging in Place" OR MH "Communities+" OR TI ("independent living" OR "home environment*" OR "aging in place" OR "ageing in place" OR community OR "community dwelling") OR AB ("independent living" OR "home environment*" OR "aging in place" OR "ageing in place" OR community OR "community dwelling")) AND (MH "Death+" OR MH "Terminal Care+" OR MH "Palliative Care" OR MH "Dignified Dying (Iowa NOC)" OR TI (Death OR "terminal care" OR "palliative care" OR dying OR "End of life" OR "well dying" OR "good death" OR "dignified death") OR AB (Death OR "terminal care" OR "palliative care" OR dying OR "End of life" OR "well dying" OR "good death" OR "dignified death")) |
| 4 | Web of Science | (Dementia* OR "Alzheimer Disease*" OR "Frontotemporal Dementia*" OR "vascular dementia*" OR "dementia, vascular" OR "Lewy body dementia") AND ("independent living" OR "home environment*" OR "aging in place" OR "ageing in place" OR community OR "community dwelling") AND (Death OR "terminal care" OR "palliative care" OR dying OR "End of life" OR "well dying" OR "good death" OR "dignified death") (Topic) |
| 5 | SCOPUS | TITLE-ABS-KEY ( ( dementia* OR "alzheimer disease*" OR "frontotemporal dementia*" OR "vascular dementia*" OR "dementia, vascular" OR "lewy body dementia" ) AND ( "independent living" OR "home environment*" OR "aging in place" OR "ageing in place" OR community OR "community dwelling" ) AND ( death OR "terminal care" OR "palliative care" OR dying OR "end of life" OR "well dying" OR "good death" OR "dignified death" ) ) |
