# Supplementary material for: Meaning and Influencing Factors of a Good Death for Community‐Dwelling Individuals With Dementia: An Integrative Review
Source: Int J Older People Nurs. 2026 Apr 12;21(3):e70078. doi: 10.1111/opn.70078 (PMC13070890; doi:10.1111/opn.70078)
Supplement: Supplementary file 2 — Table S2:‐1. Methodological quality appraisal results of qualitative studies. Table S2:‐2 Methodological quality appraisal results of quantitative non‐randomized studies. Table S2:‐3 Methodological quality appraisal results of quantitative descriptive studies. [file OPN-21-e70078-s001.docx]

**Supplementary File 2**

**Table S2-1. Methodological quality appraisal results of qualitative studies**

| **No.** | **Author (Year)** | **S1. Are there clear research questions?** | **S2. Do the collected data allow to address the research questions?** | **Qualitative** | | | | |
| --- | --- | --- | --- | --- | --- | --- | --- | --- |
|  |  |  |  | 1. Is the qualitative approach appropriate to answer the research question? | 2. Are the qualitative data collection methods adequate to address the research question? | 3. Are the findings adequately derived from the data? | 4. Is the interpretation of results sufficiently substantiated by data? | 5. Is there coherence between qualitative data sources, collection, analysis and interpretation? |
| 1 | Bolt et al (2022) | Yes | Yes | Yes | Yes | Yes | Yes | Yes |
| 2 | Bosco et al (2023) | Yes | Yes | Can't tell | Yes | Yes | Yes | Yes |
| 3 | de Jong et al (2023) | Yes | Yes | Can't tell | Yes | Yes | Yes | Yes |
| 4 | Dempsey et al (2020) | Yes | Yes | Yes | Yes | Yes | Yes | Yes |
| 5 | Han et al (2019) | Yes | Yes | Can't tell | Yes | Yes | Yes | Yes |
| 6 | Hossain et al (2022) | Yes | Yes | Can't tell | Yes | Yes | Yes | Yes |
| 7 | Jennings et al (2017) | Yes | Yes | Can't tell | Yes | Yes | Yes | Yes |
| 8 | Lawrence et al (2011) | Yes | Yes | Can't tell | Yes | Yes | Yes | Yes |
| 9 | Lemos Dekker et al (2022) | Yes | Yes | Yes | Yes | Yes | Yes | Yes |
| 10 | Livingston et al (2010) | Yes | Yes | Can't tell | Yes | Yes | Yes | Yes |
| 11 | Malhotra, Hazirah et al (2021) | Yes | Yes | Can't tell | Yes | Yes | Yes | Yes |
| 12 | Malhotra, Mohamad et al (2021) | Yes | Yes | Can't tell | Yes | Yes | Yes | Yes |
| 13 | Mamun et al (2023) | Yes | Yes | Can't tell | Yes | Yes | Yes | Yes |
| 14 | McDarby et al (2023) | Yes | Yes | Can't tell | Yes | Yes | Yes | Yes |

**Table S2-2. Methodological quality appraisal results of quantitative non-randomized studies**

| **No.** | **Author (Year)** | **S1. Are there clear research questions?** | **S2. Do the collected data allow to address the research questions?** | **Quantitative non-randomized** | | | | |
| --- | --- | --- | --- | --- | --- | --- | --- | --- |
|  |  |  |  | 1. Are the participants representative of the target population? | 2. Are measurements appropriate regarding both the outcome and intervention (or exposure)? | 3. Are there complete outcome data? | 4. Are the confounders accounted for in the design and analysis? | 5. During the study period, is the intervention administered (or exposure occurred) as intended? |
| 1 | Malhotra et al (2023) | Yes | Yes | Yes | Yes | Yes | Yes | Yes |
| 2 | Mitchell et al (2004) | Yes | Yes | Yes | Yes | Yes | Yes | Yes |
| 3 | Oh et al (2023) | Yes | Yes | Yes | Yes | Yes | Yes | Yes |
| 4 | Sampson et al (2018) | Yes | Yes | No | Yes | Yes | No | Yes |
| 5 | Sternberg et al (2014) | Yes | Yes | Can't tell | Yes | Yes | Yes | Yes |
| 6 | Tay et al (2022) | Yes | Yes | Yes | Yes | Yes | Yes | Yes |

**Table S2-3. Methodological quality appraisal results of quantitative descriptive studies**

| **No.** | **Author (Year)** | **S1. Are there clear research questions?** | **S2. Do the collected data allow to address the research questions?** | **Quantitative descriptive** | | | | |
| --- | --- | --- | --- | --- | --- | --- | --- | --- |
|  |  |  |  | 1. Is the sampling strategy relevant to address the research question? | 2. Is the sample representative of the target population? | 3. Are the measurements appropriate? | 4. Is the risk of nonresponse bias low? | 5. Is the statistical analysis appropriate to answer the research question? |
| 1 | Volicer et al (2003) | Yes | Yes | Yes | Yes | Yes | No | Yes |
